# Supplementary material for: Opportunistic Infections and Efficacy Following Conversion to Belatacept-Based Therapy after Kidney Transplantation: A French Multicenter Cohort
Source: J Clin Med. 2020 Oct 28;9(11):3479. doi: 10.3390/jcm9113479 (PMC7693007; doi:10.3390/jcm9113479)
Supplement: Supplementary file 1 [file jcm-09-03479-s001.pdf]

Table S1. Comparison of data of patients from initial cohort (from Bertrand et al.) and pooled monocentric cohort.

**Characteristics**	Monocentric cohort (N=173)	Bertrand *et al.* cohort (N =280)	p-value
**Recipients' characteristics**			
Age at transplantation - *years*	50.3 ± 16	53.9 ± 15	**0.026**
Age at conversion - *years*	56.9 ± 14	56.6 ± 15	0.780
Gender: male *n (%)*	116 (67)	169 (60.3)	0.180
CMV R+ *n (%)*	78 (56.1)	165 (58.9)	0.180
First transplantation *n (%)*	128 (73.9)	236 (84.3)	**0.010**
Donors' characteristics			
Donation after death *n (%)*	123 (77.3)	260 (92.8)	**< 0.001**
Living donor *n (%)*	36 (22.6)	19 (6.7)	**< 0.001**
Characteristics at conversion			
Time between KT and belatacept conversion – months	78.7 ± 81	31.9 ± 15	**< 0.001**
eGFR at conversion – MDRD-mL/min/1.73 m^2	40.7 ± 9	26.6 ± 15	**< 0.001**
Diabetes at conversion *n* (%)	44 (25.4)	89 (31.8)	0.180
Lymphopenia at conversion *n* (%)	80 (46.2)	216 (77)	**<0.001**
Immunosuppression			
Induction therapy			
Antithymoglobulin *n* (%)	154 (89)	100 (36.4)	**<0.001**
Basiliximab *n* (%)	2 (1.1)	173 (62.9)	**<0.001**
Maintenance therapy at conversion			
Steroids *n* (%)	35 (20.2)	241 (87.8)	**<0.001**
Tacrolimus *n* (%)	162 (93.6)	201 (71.8)	**<0.001**
Tacrolimus trough concentration – ng/mL	6.5 ± 3	6.5 ± 2	0.911
Cyclosporine *n* (%)	9 (5.2)	68 (24.3)	**< 0.001**
Mycophenolate *n* (%)	150 (86.7)	246 (87.8)	0.754
mTOR inhibitors *n* (%)	15 (8.6)	29 (10.3)	0.660
Azathioprine *n* (%)	8 (4.6)	12 (6.4)	0.660

Results are expressed according to either mean ± SD or *N* (%). Missing data are not taken into account for the calculation of percentages. eGFR, estimated glomerular filtration rate; KT, kidney transplantation; mTOR, mammalian target of rapamycin; CMV, cytomegalovirus; D, donor; R, recipient.

Table S2. OPI description in patients who developed two OPI during the follow-up.

	**OPI 1**	**OPI 2**
Patient 1	CMV Chorioretinitis	Pneumocystis
Patient 2	CMV Colitis	Pneumocystis
Patient 3	Pneumocystis	CMV pneumonitis
Patient 4	Pneumocystis	CMV pneumonitis
Patient 5	Pneumocystis	CMV pneumonitis
Patient 6	Multivisceral CMV	Pneumocystis
Patient 7	CMV Pneumonitis	Brain Toxoplasma
Patient 8	Pneumocystis	EBV induced PTLD

CMV: Cytomegalovirus. PTLD: Post-transplant lymphoproliferative disease.
